# Anti-thymocyte globulin exposure in patients with diffuse cutaneous systemic sclerosis undergoing autologous haematopoietic stem cell transplantation

**DOI:** 10.1177/23971983231188232

**Published:** 2023-07-24

**Authors:** Yu-Hsiang Chiu, Anouk Drijver, Rick Admiraal, Anna van Rhenen, Stefan Nierkens, Jacob M van Laar, Julia Spierings

**Affiliations:** 1 Department of Rheumatology and Clinical Immunology, University Medical Center Utrecht, Utrecht, The Netherlands; 2 Division of Rheumatology/Immunology/Allergy, Department of Medicine, Tri-Service General Hospital, National Defense Medical Center, Taipei; 3 Princess Máxima Center for Paediatric Oncology, Utrecht, The Netherlands; 4 Department of Haematology, University Medical Center Utrecht, Utrecht, The Netherlands; 5 Centre for Translational Immunology, University Medical Center Utrecht, Utrecht, The Netherlands

**Keywords:** Systemic sclerosis, scleroderma, haematopoietic stem cell transplantation, anti-thymocyte globulin, immune reconstitution

## Abstract

**Introduction:**

Autologous haematopoietic stem cell transplantation improves event-free survival and lung function and reduces skin thickening in patients with progressive diffuse cutaneous systemic sclerosis. Anti-thymocyte globulin is a key lymphoablative constituent of conditioning protocols and is administered in a weight-based dosage. However, whether anti-thymocyte globulin exposure contributes to response to autologous haematopoietic stem cell transplantation and lymphocyte reconstitution in diffuse cutaneous systemic sclerosis patients is unknown. We aimed to explore the relationship between anti-thymocyte globulin exposure, lymphocyte reconstitution and treatment response in diffuse cutaneous systemic sclerosis patients undergoing autologous haematopoietic stem cell transplantation.

**Methods:**

A retrospective cohort of 15 diffuse cutaneous systemic sclerosis patients undergoing autologous haematopoietic stem cell transplantation was performed. Clinical characteristics and routine laboratory results were retrieved from electronic medical records. Anti-thymocyte globulin concentrations were measured in cryopreserved plasma samples at four time points (day 1 and week 1, 2 and 4) after stem cell reinfusion. Anti-thymocyte globulin exposure was estimated using a validated population pharmacokinetic model.

**Results:**

During a median follow-up of 45 months (interquartile range 19–66), 11 (73%) patients had a treatment response, and 4 (27%) were non-responders. Although all patients received the same weight-based anti-thymocyte globulin dosage, 7.5 mg/kg divided over 3 days, anti-thymocyte globulin exposure varied. Anti-thymocyte globulin exposure was higher in responders than in non-responders (163 AU*day/mL (interquartile range 153–183) and 137 AU*day/mL (interquartile range 101–149), respectively, *p* = .026). Anti-thymocyte globulin exposure was not correlated with lymphocyte reconstitution or infection rate.

**Conclusion:**

Weight-based dosing of anti-thymocyte globulin results in variable anti-thymocyte globulin exposure and treatment response across individuals.

## Introduction

Systemic sclerosis (SSc) is a rare rheumatic connective tissue disease with a prevalence of 88 per million in Western Europe.^1^ Patients with the diffuse cutaneous subset of the disease (dcSSc) have generalised skin thickening and high risk of heart, lung and/or kidney involvement. DcSSc is associated with increased morbidity and mortality. Immunomodulatory therapies, such as methotrexate (MTX), cyclophosphamide (Cy) and mycophenolate mofetil (MMF), are pivotal in slowing disease progression.^2^ Furthermore, in rapidly progressive patients, autologous haematopoietic stem cell transplantation (HSCT) has been shown to improve skin thickening, lung function and event-free survival.^3–5^ Nonetheless, HSCT is an intensive treatment with high risk of severe side effects, including fatal cardiopulmonary complications and infections.

**Figure 1.**
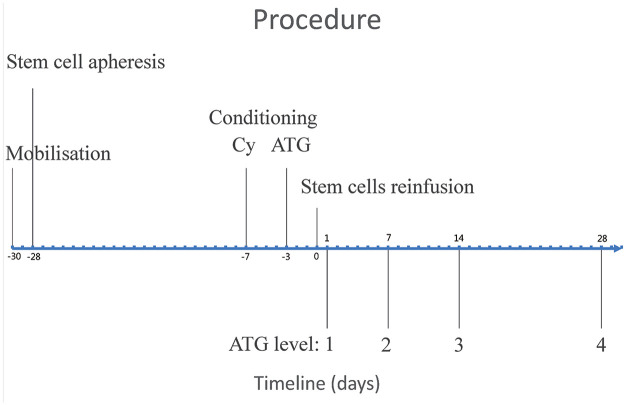
Timeline of the autologous haematopoietic stem cell transplantation and blood ATG level measurements. Cy: cyclophosphamide; ATG: anti-thymocyte globulin.

Generally, autologous HSCT consists of four steps: mobilisation, stem cell apheresis, conditioning and stem cell reinfusion (see Figure 1). Stem cells can be mobilised with Cy and granulocyte colony-stimulating factor (G-CSF). Haematopoietic stem cells are then harvested by leukapheresis. In Europe, conditioning mostly proceeds with a non-myeloablative regimen, including high-dose Cy and anti-thymocyte globulin (ATG) followed by reinfusion of CD34^+^ selected cells.^4,6,7^

ATG depletes lymphocytes primarily via complement-dependent lysis and interferes with dendritic cells and natural killer T cells.^8^ Currently, ATG dosing is based on body weight and different formulations. Patients’ blood ATG levels varied across individuals, and suboptimal exposure, including over and under, to ATG was associated with disease relapse, mortality and delayed immune reconstitution in allogeneic setting.^9,10^ Whether ATG exposure contributes to response to autologous HSCT and lymphocyte reconstitution in dcSSc patients is unknown. In this study, we explored the relationship between ATG exposure with lymphocyte reconstitution and treatment response in patients with dcSSc undergoing autologous HSCT.

## Methods

### Study design

In this retrospective cohort study, all patients with dcSSc undergoing autologous HSCT between 2014 and 2020 at the University Medical Centre Utrecht were included. Patients underwent HSCT according to the Autologous Stem Cell Transplantation International Scleroderma (ASTIS) protocol.^4^ In short, stem cells were mobilised with 4 g/m^2^ Cy divided in 2 days and filgrastim (10 μg/kg per day) and harvested with CD34 selection. The conditioning included a total of 200 mg/kg Cy over 4 days and a fixed weight-based dosage of rabbit-derived ATG (Thymoglobulin; Genzyme, Cambridge, MA, USA) 7.5 mg/kg divided over 3 days (day-3, -2, -1 relative to stem cell reinfusion on day 0) for conditioning. This study was approved by the Medical Ethical Committee Utrecht (20-285). All patients provided written informed consent.

### Data collection

Clinical characteristics and routine laboratory results were retrieved from electronic medical records. Clinical data including sex, age, disease duration at the time of HSCT, immunomodulatory treatments prior to HSCT, smoking history and pack-years, were retrieved. The duration of lymphopenia was defined as days from ATG infusion to lymphocyte counts at least 0.6 × 109/L at two consecutive measurements.

### ATG exposure

ATG concentrations were measured in cryopreserved plasma samples at four time points (day 1 and week 1, 2 and 4) after stem cell reinfusion (see Figure 1). ATG exposure was estimated using a validated population pharmacokinetic model as previously reported.^9^ Exposure was reported with the cumulative area under the concentration–time curve (AUC, AU*day/mL) from the first day of ATG-administration.

### Outcomes

Treatment response was defined as pulmonary stabilisation (with no decline more than 10% in forced vital capacity (FVC) and 15% in diffusing capacity of the lung for carbon monoxide (DLCO)) and/or skin improvement (modified Rodnan skin score (mRSS) reduction of more than 25%).^3,5^ Progressive disease was defined as increase in mRSS, deterioration of lung function with progression of interstitial lung disease (ILD) confirmed on HRCT and/or new onset of arthritis, myositis or other organ involvement, following initial treatment response. Long-term remission was defined as responder without relapse or progression of SSc during follow-up. Severity of infections was graded from 1 to 3 according to the Blood and Marrow Transplant Clinical Trials Network grading scale (see Supplementary Table S1).^11^

### Statistical analysis

Categorical data were presented in numbers with percentages, and numeric data were presented in median and interquartile range (IQR). Differences between groups were examined with Wilcoxon rank-sum test for contentious variables and Fisher’s exact test for categorical variables. A *p*-value < 0.05 was considered statistically significant. All statistics were performed in R 4.0.3.

## Results

### Patient characteristics

Fifteen patients were included in this study. Seven (47%) were female, and median age was 43 years (IQR 37–50). The median disease duration at HSCT was 7 months (IQR 4–17). Nine patients (60%) were anti-topoisomerase I positive, two (13%) had anti-RNA polymerase III antibodies and three (20%) had other autoantibodies. All patients had diffuse skin involvement and gastrointestinal disease (mainly gastroesophageal reflux disease), 11 (73%) patients had ILD, 7 (47%) had cardiac involvement (defined as fibrotic myocardial changes seen on magnetic resonance imaging). Patients with pulmonary arterial hypertension were excluded from HSCT. Eight (53%) patients were past smokers with a median of 15 pack-years (IQR 6–35). Six patients (40%) used MTX, five (33%) MMF, two (13%) Cy and one (7%) azathioprine prior to HSCT. Four (27%) patients were DMARD naïve before the start of HSCT. The median follow-up was 45 months (IQR 19–66). The median reinfused CD34^+^ stem cells were 4.26 (IQR 3.95–5.01) × 106/kg (see Table 1).

**Table 1.** Patient characteristics.

Patient characteristics	*n* = 15
Age	43 (37–50)
Female, ***n*** (%)	7 (47)
Weight (kg)	70 (64–74)
DMARDs history, ***n*** (%)	
MTX	6 (40)
MMF	5 (33)
Cy	2 (13)
Azathioprine	1 (7)
⩾ 2 DMARDs history	3 (20)
Disease duration at HSCT, months	7 (4–17)
mRSS	25 (19–28)
SSc-associated major organ involvement, ***n*** (%)	
ILD	11 (73)
Cardiac involvement	7 (47)
PAH	0
FVC % of predicted	83 (68–95)
DLCO % of predicted	62 (54–73)
Ever smoker, *n* (%)	8 (53)
ATA positive, *n* (%)	9 (60)
ARA positive, *n* (%)	2 (13)
Reinfused stem cells (106 cells/kg)	4.26 (3.95–5.01)

DMARDs: disease-modifying anti-rheumatic drugs; MTX: methotrexate; MMF: mycophenolate mofetil; Cy: cyclophosphamide; mRSS: modified Rodnan skin score; SSc: systemic sclerosis; ILD: interstitial lung disease; PAH: pulmonary arterial hypertension; FVC: forced vital capacity; DLCO: diffusing capacity of the lung for carbon monoxide; ATA: anti-topoisomerase I antibodies; ARA: anti-RNA polymerase III positive.

Data are presented in median (interquartile range (IQR)) or *n* (%).

### Treatment response

Eleven patients (73%) had an initial response to HSCT. Long-term remission was achieved in eight (53%) patients, and three initial responders relapsed and had progressive disease during follow-up, at a median time of 8 months (IQR 5–17) post-HSCT. Patients with progressive disease after initial response, presented with skin and/or lung progression and new onset myositis, were all well-controlled with immunosuppressive treatments. Four patients (27%) had progressive disease and did not respond to HSCT. At 1 year after HSCT, responders had a median change in mRSS –14 (IQR −18 to −8), FVC 8% of predicted (IQR −1 to 15) and DLCO 5% of predicted (IQR 2–14); non-responder had a median change in mRSS –1 (IQR –2 to 3), FVC –14% of predicted (IQR −19 to −8) and DLCO –14% of predicted (IQR −17 to −10). The changes in mRSS, FVC and DLCO at 1 year were not correlated with ATG exposure. There was one treatment-related mortality; the patient developed thrombotic microangiopathy 3 days after stem cell reinfusion and deceased two and a half months thereafter.

### ATG exposure and lymphocyte reconstitution

Although all patients received a fixed weight-based ATG dosage, ATG exposure varied (see Table 2). The median ATG exposure was 153 AU*day/mL (IQR 148–180). ATG exposure was higher in responders (see Supplementary Figure S1), 163 AU*day/mL (IQR 153–183), than in non-responders, 137 AU*day/mL (IQR 101–149), *p* = .026. Lymphocyte counts normalised to more than 0.6 × 109/L in a median time of 29 days (IQR 22–42). There was no significant difference in duration of lymphopenia between responders and non-responders. The depth and duration of lymphoablation were not correlated with ATG exposure. The reconstruction of neutrophil and platelet was not correlated with the ATG exposure either. The reconstitution of lymphocytes, platelets and neutrophils was shown in Supplementary Figure S2.

**Table 2.** ATG-exposure comparison on outcomes.

Outcomes	ATG exposure	*p*-value
		.945
With viral infection	156 (149–179)	
Without viral infection	153 (117–191)	
		.026
Responders	163 (153–183)	
Non-responders	137 (101–149)	
		.536
Progressive disease following initial treatment response	178 (164–180)	
Response with long-term remission	153 (141–168)	

ATG: anti-thymocyte globulin.

The median (interquartile range) ATG exposure was presented in the cumulative area under the concentration–time curves (AU*day/mL).

### Viral infection

Four (27%) patients had a symptomatic viral infection and three had more than one viral infections. Cytomegalovirus (CMV) reactivation (all grade 2) occurred in four (27%), Epstein-Barr virus (EBV) reactivation in two (13%; grade 2 and grade 3) and BK virus (grade 2) in one patient.

Patients with symptomatic viral infections (⩾ grade 2) had higher lymphocyte count before ATG was initiated (median 0.96 × 109/L (IQR 0.87–1.14) versus 0.51 × 109/L (0.42–0.68), *p* = .016) and greater drop in lymphocytes after ATG administration, median 0.92 × 109/L (IQR 0.84–1.10) versus 0.48 × 109/L (IQR 0.40–0.64), *p* = .010 (see Supplementary Figure S3). Lymphocyte reconstitution rate and ATG exposure were not associated with high-grade occurrence of symptomatic viral infection (⩾ grade 2).

Seven (47%) patients had an asymptomatic increase in viral load (viral infection grade 1) during routine monitoring not requiring intervention. Blood lymphocyte counts before ATG infusion, lymphocyte reconstitution rate and ATG exposure did not significantly differ between patients with and without viral infection.

## Discussion

In this study, we explored the relationship between ATG exposure, lymphocyte reconstitution and clinical outcomes in patients with dcSSc undergoing autologous HSCT. Although all patients received the same weight-based ATG dosage, ATG exposure varied. This finding is in line with studies investigating ATG exposure in haematological patients in the context of allogeneic HSCT.^9,10,12^ A possible explanation for the variation in ATG exposure is that the volume of distribution and clearance of ATG are influenced by weight and lymphocyte count over time;^12^ the underlying disease and HSCT protocol, including conditioning regimen and source of stem cells may also contribute to these variety. In our patients, the fluctuations in blood ATG level also revealed a non-linear correlation with weight and lymphocyte count, which contributed to the final estimated ATG exposure.

In this study, ATG exposure was higher in HSCT responders than non-responders. However, the depth and duration of lymphoablation in blood was not correlated with ATG exposure or treatment response. In studies investigating allogeneic HSCT, high ATG exposure was associated with less graft-versus-host-disease (GvHD) but higher risks of treatment-related mortality and infection.^9,13^

ATG may have effects other than lympho-depletion. Cell surface markers are non-exclusively presented on T cells and also on B cells, natural killer cells and other antigen presenting cells.^8^ A down-regulation of β2 integrin leukocyte function-associated antigen-1 was found on lymphocytes, monocytes and neutrophils with a dose-dependent effect of ATG.^14^ Although ATG does not influence macropinocytosis and receptor-mediated endocytosis in dendritic cells (DCs), ATG interferes with DCs via complement-mediated DC lysis, DC–T-cell interaction blockade and monocyte-derived DCs maturation inhibition.^15,16^ We did not study the effects of ATG on the innate immune system, and this could be interesting for future studies.

A previous study that explored ATG-free conditioning in autologous HSCT for autoimmune diseases reported lower complication rates compared to European Group for Blood and Marrow Transplantation (EBMT) registry on autoimmune diseases, maybe due to reduced lymphoablation;^17^ however, relapse-free survival was also lower. In our study, the higher ATG exposure in responders suggested that ATG administration contributed to disease control in dcSSc. Also, no delayed lymphocyte reconstitution or increased infection rate was observed in patients with higher ATG exposure. ATG exposure may correlate with treatment response due to immunomodulatory effect and lymphoablation not only in blood but also in tissues and lymph nodes, which cannot be detected simply by blood cell count, whereas blood lymphocyte count and duration of lymphopenia correlated more directly with infectious complications. In our cohort, the occurrence of symptomatic viral infections after stem cell transplantation was 27%, which is in line with reported infectious events in the ASTIS trial.^4^ Due to routine monitoring of viral load, asymptomatic viral infections (grade 1) not requiring treatment were detected.

Interestingly, patients with symptomatic viral infections (⩾ grade 2) had higher lymphocyte count prior to ATG administration and therefore greater reduction in lymphocyte count after ATG infusion (see Supplementary Figure S2). It is possible that patients with higher lymphocyte count in homeostasis are more vulnerable to viral infection during lymphoablation in HSCT. In a study with allogeneic HSCT, delayed lymphocyte reconstitution was associated with CMV reactivation;^18^ similarly, the patients with CMV reactivation had higher baseline lymphocyte count. Although these ideas are attractive, our study cannot directly explore this mechanism. At this stage, such concepts remain speculative. Moreover, the reconstitution of lymphocyte subsets, especially T cells, contributes to infection vulnerability and side effects in its degree.^19^ Reconstitution of CD4^+^ T cells to more than 5 × 107/L within 100 days was associated with survival, GvHD and viral reactivation in allogeneic HSCT, and was achieved with individualised ATG dosing.^20^ Unfortunately, we were unable to further differentiate the lymphocyte subsets in this study.

All patients in our study received HSCT with CD34^+^-cell selection. CD34^+^-cells selection in HSCT is intended to prevent reinfusion of autoreactive lymphocytes and foster the self-tolerance in the immune reconstitution process. In a Japanese post hoc analysis, patients who received CD34-selected cells had greater improvement in FVC and higher long-term remission than patients who received non-CD34-selected stem cells.^21^ Furthermore, a recently published analysis of the EBMT registry for SSc patients undergoing autologous HSCT also revealed that HSCT with CD34-selection was associated with higher treatment response rate and did not increase infectious complication.^7^

To our knowledge, our study is the first to investigate the impact of ATG exposure on outcomes of SSc patients undergoing HSCT. A strength of our study is the long follow-up and the use of a validated pharmacokinetic model. However, our study has some limitations. The proportion of patients with long-term remission (53%) was lower than which in the ASTIS trial (4 years event-free survival 81%) and in a recently published Brazilian cohort (5 years event-free survival 71.8%).^4,22^ Because of the low prevalence of dcSSc, the sample size is small. Due to the retrospective design, we could only evaluate ATG concentration in plasma collected at four time points. Still, the population pharmacokinetic study in patients undergoing allogenic HSCT revealed that weight and lymphocyte count before ATG infusion were associated with ATG clearance, and ATG exposure was strongly associated with patients’ outcome.^9,12^ Monitoring ATG pharmacokinetic and lymphocyte count with subsets differential count may help to identify optimal dosing of ATG in SSc patients undergoing autologous HSCT. An intensified prospective blood ATG concentration measurement would provide a more accurate ATG exposure estimation, which is included in the ongoing clinical UPSIDE trial (NCT04464434) investigating first-line autologous HSCT in early SSc patients.^23^

In conclusion, our study showed that ATG exposure in transplanted SSc patients varied among the same weight-based dosage and was correlated with treatment response.

## Supplemental Material

sj-pdf-1-jso-10.1177_23971983231188232 – Supplemental material for Anti-thymocyte globulin exposure in patients with diffuse cutaneous systemic sclerosis undergoing autologous haematopoietic stem cell transplantation Click here for additional data file. Supplemental material, sj-pdf-1-jso-10.1177_23971983231188232 for Anti-thymocyte globulin exposure in patients with diffuse cutaneous systemic sclerosis undergoing autologous haematopoietic stem cell transplantation by Yu-Hsiang Chiu, Anouk Drijver, Rick Admiraal, Anna van Rhenen, Stefan Nierkens, Jacob M van Laar and Julia Spierings in Journal of Scleroderma and Related Disorders
