# Supplementary material for: Anti-thymocyte globulin exposure in patients with diffuse cutaneous systemic sclerosis undergoing autologous haematopoietic stem cell transplantation
Source: J Scleroderma Relat Disord. 2023 Jul 24;8(3):241–6. doi: 10.1177/23971983231188232 (PMC10515999; doi:10.1177/23971983231188232)
Supplement: sj-pdf-1-jso-10.1177_23971983231188232 – Supplemental material for Anti-thymocyte globulin exposure in patients with diffuse cutaneous systemic sclerosis undergoing autologous haematopoietic stem cell transplantation [file sj-pdf-1-jso-10.1177_23971983231188232.pdf]

Table S1. Viral infection grading

	**Grade 1**	**Grade 2**	**Grade 3**
EBV	EBV reactivation not treated with rituximab	EBV reactivation requiring institution of therapy with rituximab	EBV PTLD
CMV	Asymptomatic CMV viremia untreated or a CMV viremia with viral load decline by at least 2/3 of the baseline value after 2 weeks of therapy	Clinically active CMV infection (e.g. symptoms, cytopenia) or CMV Viremia not decreasing by at least 2/3 of the baseline value after 2 weeks of therapy	CMV end-organ involvement (pneumonitis, enteritis, retinitis)
BKV	BK viremia or viruria with cystitis not requiring intervention	BK viremia or viruia with clinical consequence requiring prolonged therapy and/or surgical intervention	

Adapt from the Blood and Marrow Transplant Clinical Trials Network Technical Manual of Procedures. Abbreviations: Epstein-Barr virus (EBV); cytomegalovirus (CMV); BK virus (BKV).

Table S2. Individual case data

Patients	DMARDs history	Weight (kg)	ATG exposure (AU*day/mL)	EBV	CMV	BK
1	Naive	66	183.41	1	0	0
2	MMF	60	182.90	0	0	0
3	MTX	64	177.53	0	0	1
4	MTX, Cy	64	163.39	0	0	1
5	Cy	74	149.22	0	0	1
6	AZA	36	33.60	0	0	1
7	Naive	83	149.80	1	0	0
8	MTX	70	229.12	0	0	0
9	Naive	70	153.30	0	0	0
10	MMF	78	152.25	3	2	0
11	MMF	71	160.28	1	2	2
12	MMF, MTX	84	123.83	1	0	0
13	MTX	72	187.57	2	2	0
14	MMF, MTX	73	147.36	0	2	0
15	Naive	62	80.00	0	0	0

The severity of viral infection was graded according to the Blood and Marrow Transplant Clinical Trials Network Technical Manual of Procedures.
Abbreviations: disease modifying anti-rheumatic drugs (DMARDs), methotrexate (MTX), mycophenolate mofetil (MMF), cyclophosphamide (Cy), azathioprine (AZA).

Figure S1.

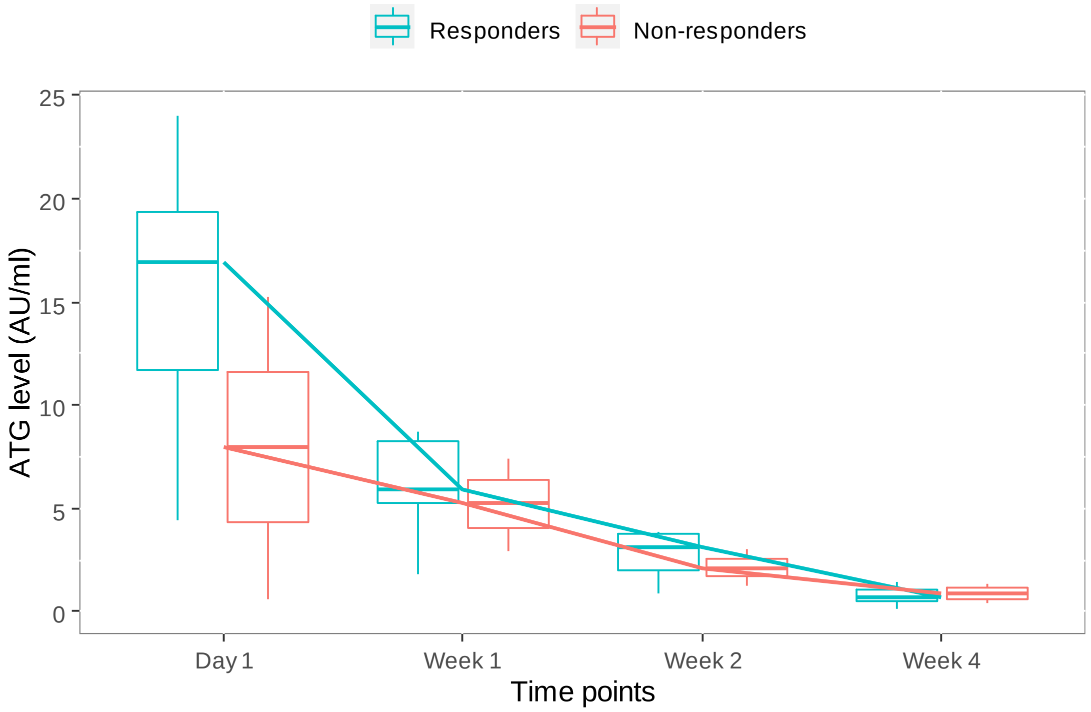

Figure S1. Plasma anti-thymocyte globulin (ATG) levels at four time points after stem cell reinfusion and grouped by treatment response to autologous haematopoietic stem cell transplantation.

Figure S2.

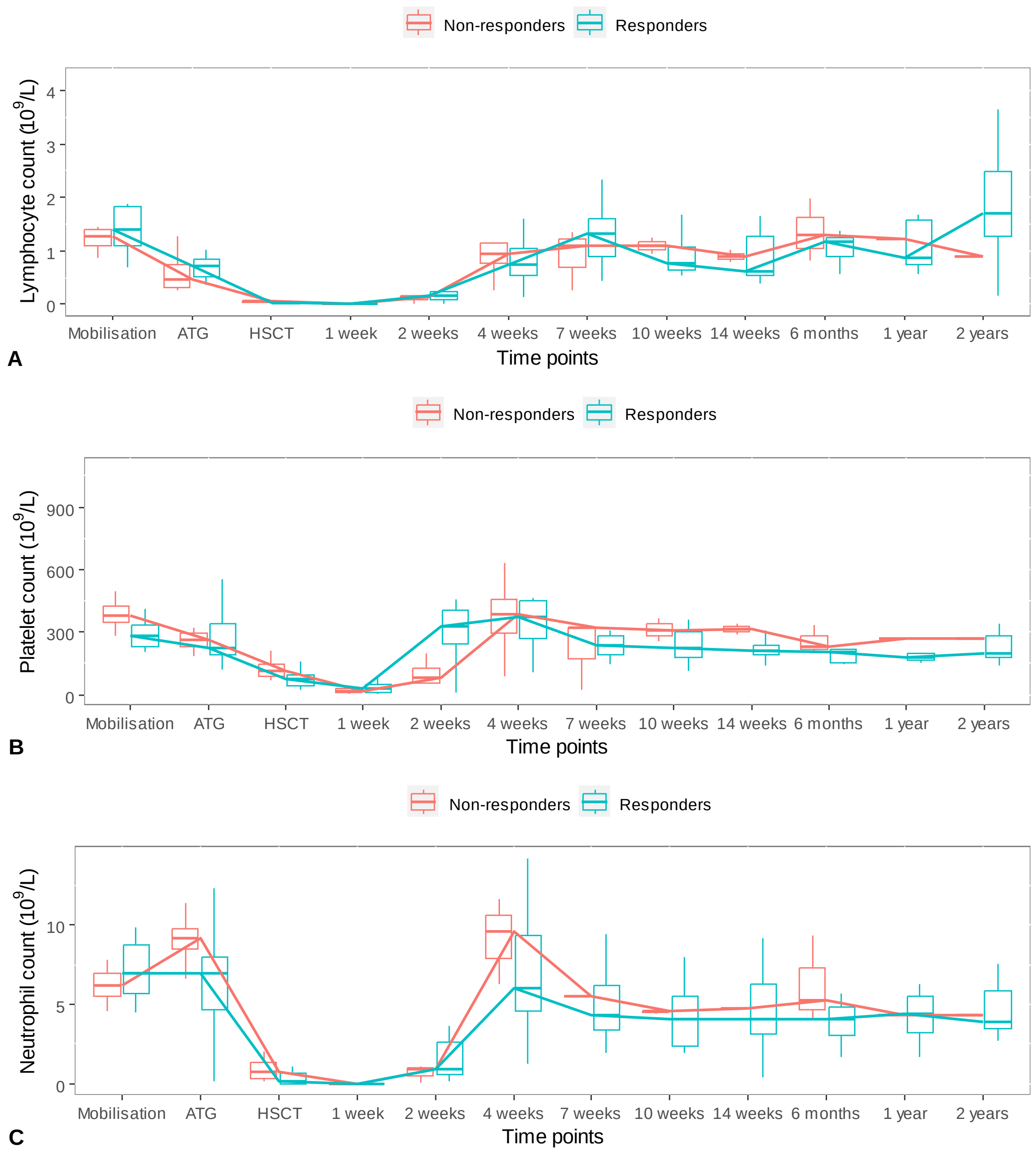

Figure S2. Changes of lymphocyte count, platelet count and neutrophil over time grouped by treatment response to autologous haematopoietic stem cell transplantation.

Figure S3.

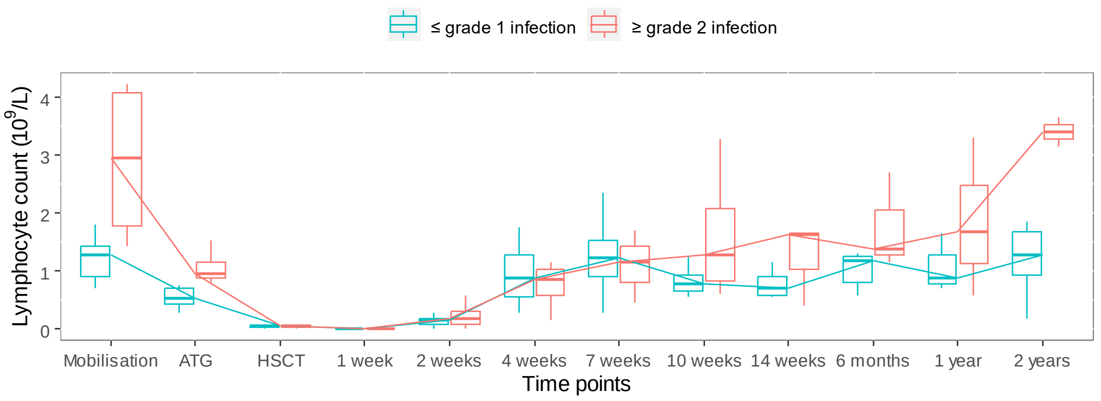

Figure S3. Changes of lymphocyte count over time grouped by viral infection grade (≥ grade 2, symptomatic infection according to the Blood and Marrow Transplant Clinical Trials Network Technical Manual of Procedures.)
